# Treatment of psychiatric comorbidities and interaction patterns in Coffin‐Siris syndrome: A case report of a 4‐year‐old girl

**DOI:** 10.1002/ccr3.8230

**Published:** 2024-02-02

**Authors:** Ann‐Christin Jahnke‐Majorkovits, Christine Fauth, Manuela Gander, Kathrin Sevecke

**Affiliations:** ^1^ A.ö. Landeskrankenhaus Hall, Kinder‐ und Jugendpsychiatrie Psychotherapie und Psychosomatik Hall in Tirol Austria; ^2^ Universitätsklinik für Kinder‐ und Jugendpsychiatrie Medizinische Universität Innsbruck Innsbruck Austria; ^3^ Institut für Humangenetik Med. Univ. Innsbruck Innsbruck Austria; ^4^ Institute of Psychology University of Innsbruck Innsbruck Austria

**Keywords:** Coffin‐Siris syndrome, interaction patterns, psychiatric comorbidities, therapeutic intervention

## Abstract

Coffin‐Siris syndrome (CSS) is a rare genetic disorder and often co‐occurs with attention‐deficit hyperactivity disorder (ADHD) and autism spectrum (ASD). The present case study illustrates possible therapeutic interventions of these common psychiatric comorbidities taking into account the family interaction patterns. This can contribute to improve holistic management and overall level of functionality.

## 1 INTRODUCTION

Coffin‐Siris syndrome (CSS) is a rare clinically and genetically heterogeneous disorder, mostly caused by mutations in the genes encoding subunits of the BAF complex, including *ARID1A*, *ARID1B*, *ARID2*, *DPF2*, *SMARCA4*, *SMARCB1*, *SMARCC2*, *SMARCE1*, *SOX11*, and *SOX4*.^1^, ^2^, ^3^, ^4^ Up to now, around 200 cases have been described. Main characteristic features are hypoplasia or aplasia of the nail or the distal phalanx of the pinkie finger (“fifth‐digit syndrome”), coarse facial features, and moderate‐to‐severe intellectual disability.^5^, ^6^, ^7^, ^8^, ^9^ Characteristic features of the face are thick eyebrows, long eyelashes, broad bridge of the nose, wide mouth with thick, everted upper and lower lip, and abnormal position or shape of the ears.^3^, ^4^, ^9^ Common anomalies include failure to thrive, feeding problems, short stature, microcephaly, ocular symptoms (cataract, ptosis, strabismus), cardiac anomalies, hypertrichosis (arms, face, back), and sparse scalp hair.^4^, ^9^

Autism spectrum disorder (ASD) is also assumed to have a strong genetic basis and is genetically highly heterogeneous with risk contribution from hundreds of genes. One of these ASD risk genes is *ARID1B*.^10^, ^11^, ^12^, ^13^ As *ARID1B* haploinsufficiency is a common cause of intellectual disability (ID), autism spectrum disorder (ASD), and emotional disturbance, interference caused by *ARID1B* are better described as a spectrum. This includes non‐syndromic ID and CSS cases under the generic term *ARID1B*‐associated disorders (*ARID1B*‐RD) with or without additional physical and/or neurological symptoms.^13^ While the overall prevalence is unknown, individuals with *ARID1B*‐related disorders also suffer from an increased risk of attention‐deficit/hyperactivity disorder (ADHD) diagnoses^12^, ^13^ The ARID1B gene thus plays an essential role in normal brain development and behavior.^12^

Since the association between *ARID1B* and CSS, ASD, ADHD, and ID is well established, there are already a few reports which focus on psychiatric comorbidities and their assessment. Swillen et al.^14^ report compulsive interests, a strong dependence on patterns and rituals, and unusual fears as characteristic behavioral problems of 12 children and adolescents with CSS. Lohiya^15^ points out the need for an assessment of comorbidities related to neurocognition, behavior, and socio‐adaptive functioning in CSS in the case of a child with CSS and Autism due to an *ARID1B* mutation. Krause and Rose^16^ describe the case of a 12‐year‐old patient with CSS with additional neuropsychological evaluations that revealed a diagnosis of a specific learning disorder, a developmental coordination disorder and a language disorder. However, there are no descriptions of a specific psychological‐therapeutic approach into which the diagnostic results are integrated. This is the topic of this case report. We describe the specific therapeutic interventions after the diagnosis and the influence of the interaction patterns between the patient and her mother on the overall symptoms and level of function.

## 2 CASE DESCRIPTION

The 4‐year‐old patient with CSS was referred to the parent–child unit by a pediatrician.

The girl was diagnosed with CSS at the age of 2 years and 2 months. She showed characteristic clinical findings including a low frontal hairline, thick eyebrows, a wide nasal bridge, down‐slanted palpebral fissures, a broad tip of nose, a thin vermillion of the upper lip and a thick everted vermillion of the lower lip, deep‐set protruding ears and hypoplastic nails on the fifth fingers and toes and a significantly delayed development. The girl had started to walk at the age of 23 months and there was no active language apart from sounds such as “Aa” or “Da.” Listening to loud music or loud clapping, the child may glance for a moment, but did not react to any noises, including very loud ones.

Diagnosis of CSS was confirmed by genetic testing which revealed a pathogenic de novo frameshift variant in the *ARID1B* gene [NM_020732.3, variant c.1150_1151delGG; Coffin‐Siris syndrome 1, MIM #135900].

The mother described that her everyday life was strongly affected by her child's needs. For example, her daughter needed certain music on the cell phone all the time, pieces of cloth and an object to “suck.” These could also be used to calm her down or to perform activities she did not like (e.g., diapering, bathing). The daily routine only worked with the help of music, for example, brushing teeth and changing diapers. Whenever there were many people and noises, such as birthday parties or shopping, the child reacted very stressed and vomited very often. This was also the reason why the family hardly ever left the house with their child. When riding a bicycle, the patient only accepted a certain path. When the mother tried to turn off the music or to take different routes, her daughter began hurting herself (biting her lip bloody, pulling out her hair, and sometimes eating it).

The trichotillomania had improved significantly since a complete shave, but the girl now reacted to tension or frustration with strong bobbing. In addition, eating was also very difficult, she only ate certain foods (spaghetti, watermelon, pretzels, bread with cheese). During the night, there were always longer periods of wakefulness of up to 5 h.

The patient lives together with the parents and two half‐siblings (15 and 16 years old). There had been no kindergarten visit so far, the mother was very concerned that she might not feel well, that other children could annoy her or that the teachers could not respond well enough to her. Therefore, the mother preferred exclusive care by herself. However, she felt very exhausted for this reason and sad and drained on some days. The patient already received early support for 2 h a week as well as speech therapy and physiotherapy at home.

In addition to the diagnostic clarification, the main treatment goals consisted in coping with everyday situations (promoting more flexibility, reducing listening to music, expressing hunger and thirst).

## 3 RESULTS

### 3.1 Psychological assessment

The psychiatric diagnosis revealed the common psychiatric comorbidities in form of enduring developmental problems, somatic complaints, and withdrawal behavior (Child Behavior Checklist (CBCL 1½–5)^17^ as well as an atypical autism (ADOS‐2)^18^; (M‐CHAT).^19^ In addition, attachment patterns of the mother were classified using the Adult Attachment Projective Picture System (AAP)^20^ in order to assess personal relationship experiences and unconscious attachment‐related defense mechanisms. For this purpose, the mother was asked to tell a story for each of the seven pictures of the AAP using a set of standardized questions: What happens on the picture? What are people thinking or feeling? What might happen next? All narratives were transcribed verbatim and assessed by two independent reliable raters. The narratives were evaluated with regard to the following aspects: (1) Influence of personal experiences, (2) representation of attachment‐related resources in the stories: does the person portrayed have an internalized secure basis (willingness to engage in self‐reflection, haven of safety) or do they experience themselves as effective in their actions (self‐efficacy), (3) connection to other people (connectedness), (4) formation of dyadic relationships (synchrony). In addition, three defense strategies were assessed: deactivation, cognitive disconnection (ambivalence), and segregated systems. Based on these criteria, individuals can be classified with a secure or an insecure attachment pattern (avoidant, preoccupied, unresolved trauma).

In the present case, the independent double rating of the AAP revealed a preoccupied attachment status (E). Individuals with this attachment representation are unable to tolerate the intensity of feelings of sadness and aloneness and experience “cognitive disconnection”. This is expressed as a distorted approach to attachment‐related issues in the form of conflicting, contrasting images and judgments. The sequences of events appear to be split off or conflictual. This includes a basic insecurity, the expression of conflict and anger as well as stories without a “red thread.”

These aspects of an insecure‐preoccupied representation of attachment were mainly caused by the insecurity (bench: “*either it is a child and has problems with its parents or with school, or it is lovesick, or it is a child who is impaired and is totally sad because it doesn't feel accepted, doesn't belong and doesn't feel being understood*”, frequent expressions of “*I don't know*”), a missing internalized secure base (bench: “*I actually only see sadness, despair, sad, helpless…yes…alone its just the whole package”*) and the main character's lack of capacity to act in the narratives. In the individual stories, references to own personal experiences were repeatedly made, but attachment‐related resources also became clear in the dyadic images in the form of synchrony.

### 3.2 Treatment

The multimodal inpatient treatment included occupational therapy, speech therapy, physiotherapy, comprehensive nutritional advice, and psychological counseling with video‐supported, interaction‐centered sessions for 3 weeks. As part of a gentle, graduated exposition, the mother was instructed on how to reduce the constant listening to music and how to separate from her child for a limited time by establishing a reliable ritual (announcement, saying goodbye, reunion). This enabled the mother to switch off the music at moments when her child was happily occupied with other things. The mother was also taught how to enable her child's exploration of the area independently by only staying within sight. Further psychoeducation on the importance of exploration and autonomy in child development supported this process. In the biographical work, the mother's own relationship experiences were related to her current parenting behavior. The mother became aware that she was compensating her own experiences of a lack of emotional support by overprotecting her own child. The experiences in the protected treatment setting helped to reduce these worries and to gain confidence in other people as trustworthy caregivers for her child. In order to relieve the mother in the care, the father was also invited to join treatment. He refused due to own fears and social inhibitions.

Following inpatient treatment, our patient was discharged into an outpatient treatment setting with regular child psychiatric and pediatric check‐ups. 3 months after discharge, the mother reported further progress in development. Her child showed new kinds of behavior, for example, by bringing a pot to the mother while cooking and pushing people away when there is no desire for contact. In addition, the mother noticed that her child could now express emotions such as sadness in form of crying. In the kindergarten, careful integration began while the mother was present. In this environment, the girl primarily used a small playhouse as a place of refuge from which she observed other children and explored when she felt safe enough. As agreed, the mother started her own psychotherapy in order to (further) reflect her fears and insecurities. The occupational therapy and speech therapy took place in an external environment in order to enable new experiences for her child.

## 4 DISCUSSION AND CONCLUSION

The case of a 4‐year‐old girl with Coffin‐Siris syndrome caused by a pathogenic de novo frameshift variant in the *ARID1B* gene was described. The child's psychiatric diagnosis revealed a developmental delay in all areas and an atypical autism with symptoms of a lack of social interaction, no active language and rigid and repetitive behavioral patterns. These findings match the already known phenotypes of *ARID1B*‐related disorders. In a study of 143 patients with an *ARID1B* spectrum, it could be shown that both patients with an *ARID1B* CSS and with an *ARID1B* ID showed autistic traits in over 55% of the cases.^11^ Over 83% of the patients in both groups showed behavioral problems in form of poor sociability (19.7%), obsessive (15.5%), and rigid behavior (8.5%).^11^ Thus, the role of the *ARID1B* gene in normal brain development and behavior becomes obvious.^12^

These aspects can contribute to the understanding of the CSS‐related symptoms and should be taken into account when planning appropriate therapeutic measures for affected children. Additionally, we described the concrete therapeutic interventions in which family and interaction‐related aspects were included. In the case described above, the psychological testing revealed the mother's tendency to social isolation, fears, and overprotection. The assessment of attachment patterns with the AAP turned out to be very helpful to better understand the dysfunctional interaction patterns between mother and daughter and to relate them to the mother's own biographical experiences. The AAP is a suitable instrument for the assessment of attachment representations and integration into psychotherapy.^21^, ^22^, ^23^, ^24^ The mother's difficulty in tolerating feelings such as sadness and loneliness manifested in the interaction with her daughter in overprotection and a lack of autonomy. As a result, the daughter's developmental delay as part of the CSS was presumably additionally intensified.

By becoming aware of and working through the mother's inner working models, which Bowlby (1980) regards as a core task of the therapeutic process,^25^ it was possible to talk about the importance of the kindergarten and an own psychotherapy. Without the multimodal child psychiatric treatment, there would have been a higher risk that the symptoms of the developmental delay in the context of CSS would have been aggravated by social isolation and a lack of contact with other children. The mother's exhaustion illustrates the need to be supported by a comprehensive system of helpers (outpatient family support, psychological support, involvement of other caregivers, social integration in kindergartens and schools) of CSS‐affected children.

Therefore, it is useful not only to diagnose the known psychiatric comorbidities in affected children but also to treat them specifically—as the present case study shows—also taking into account the family interaction patterns since the diagnosis of CSS is associated with lifelong restrictions and a high level of care. The cooperation of a multi‐professional team and a multimodal treatment should be initiated. As a result, the holistic functional level of children with CSS can be improved, as well as their quality of life. This may also be the subject of future research in this area.

## AUTHOR CONTRIBUTIONS

**Ann‐Christin Jahnke‐Majorkovits:** Conceptualization; writing – original draft. **Christine Fauth:** Writing – review and editing. **Manuela Gander:** Supervision. **Kathrin Sevecke:** Conceptualization; supervision.

## CONFLICT OF INTEREST STATEMENT

No conflicts of interest.

## FUNDING INFORMATION

The author's received no funding for the research, authorship and publication of this article.

## CONSENT

Written informed consent was obtained from the patient to publish this report in accordance with the journal's patient consent policy.

## Data Availability

The data that support the findings of this study are available on request from the corresponding author. The data are not publicly available due to privacy or ethical restrictions.
